# Pancreatic Juice Cytology Evaluations Using Synthetic Secretin and Serial Pancreatic Juice Aspiration Cytological Examination for the Diagnosis of Pancreatic Ductal Adenocarcinoma

**DOI:** 10.3390/diagnostics13091536

**Published:** 2023-04-25

**Authors:** Yohei Takeda, Kazuya Matsumoto, Takumi Onoyama, Taro Yamashita, Hiroki Koda, Wataru Hamamoto, Yuri Sakamoto, Takuya Shimosaka, Shiho Kawahara, Yuta Seki, Hiroki Kurumi, Yasushi Horie, Hajime Isomoto, Naoyuki Yamaguchi

**Affiliations:** 1 Division of Gastroenterology and Nephrology, Department of Multidisciplinary Internal Medicine, Faculty of Medicine, Tottori University, Yonago 683-8504, Japan; 2 Division of Organ Pathology, Department of Pathology, Faculty of Medicine, Tottori University, Tottori 683-8504, Japan; 3 Department of Gastroenterology and Hepatology, Graduate School of Biological Sciences, Nagasaki University, 1-7-1 Sakamoto, Nagasaki 852-8501, Japan

**Keywords:** pancreatic ductal adenocarcinoma, pancreatic juice cytology, synthetic secretin, serial pancreatic juice aspiration cytological examination

## Abstract

Pathological examination by endoscopic ultrasound–fine needle aspiration is not possible in approximately 10% of pancreatic tumor cases. Pancreatic juice cytology (PJC) is considered an alternative diagnostic method. However, its diagnostic capability is insufficient, and PJC has been repeatedly redevised. Serial pancreatic juice aspiration cytological examination (SPACE) and secretin-loaded PJC (S-PJC) have been recently introduced as alternative diagnostic methods. This study aimed to determine the diagnostic capacity and safety of SPACE and S-PJC using a propensity score-matched analysis. The sensitivity, specificity, and accuracy were 75.0%, 100%, and 92.3% for S-PJC, respectively, and 71.4%, 100%, and 92.3% for SPACE, respectively, meaning that there was no significant difference between the groups. Four patients (15.4%) each in the S-PJC and SPACE groups experienced complications, including postendoscopic retrograde cholangiopancreatography, pancreatitis, and cholangitis. Overall, there was no difference in efficacy and safety between the SPACE and S-PJC groups.

## 1. Introduction

Pancreatic ductal adenocarcinoma (PDAC) is often unresectable at the time of diagnosis. Thus, chemotherapy is the recommended treatment in many cases. A pathological diagnosis is critical for selecting the optimal chemotherapy regimen, with endoscopic ultrasound–fine needle aspiration (EUS-FNA) being the most widely used pathological diagnostic method. However, it has a sensitivity of 85–93% [1,2,3] and fails to diagnose PDAC in approximately 10% of cases. Thus, EUS-FNA is not yet a completely recognized diagnostic method, although various innovations have been made in terms of needle size and tip shape. The main reasons for diagnosis failure include interruption of the puncture route by blood vessels or the main pancreatic duct, inability to perform tumor imaging, and difficulty in withdrawal of anticoagulants.

Currently, pancreatic juice cytology (PJC), another pathological diagnostic method, is increasingly introduced as an alternative to EUS-FNA when the latter is too difficult to perform. However, its sensitivity is also inadequate, with a PDAC diagnosis rate of 46.7–76.4% [4]. Diagnosis of PDAC by cytology was first reported using duodenal fluid collected using a tube [5,6]. Subsequently, the current PJC, in which a duodenal fiberscope is inserted, and a catheter is used under direct vision to transpapillarily aspirate pancreatic juice from the pancreatic duct, was reported [7]. There have been various attempts to improve these diagnostic methods, and some techniques include brushing and washing cytology, endoscopic nasopancreatic drainage (ENPD), and secretin-loaded PJC (S-PJC) [8]. Brush cytology is a method in which a brush is used to scrape the stenosis of the pancreatic duct and collect the cells [4]. The method of scraping the stenosis with only a guidewire has also been reported [9]. Cytology using a double- or triple-lumen catheter placed in the pancreatic duct stenosis and washed with saline to collect a wash containing pancreatic juice is another alternative. This cytological diagnosis with pancreatic duct lavage fluid is useful for collecting highly viscous pancreatic juice and has been reported to be used for the diagnosis of malignant intraductal papillary mucinous neoplasm (IPMN) [10]. In 2012, Iiboshi et al. reported the usefulness of multiple consecutive PJC examinations with ENPD [11], prompting the development of serial pancreatic juice aspiration cytologic examination (SPACE).

S-PJC with porcine secretin was once a common detection method but is no longer used due to concerns of infection. Secretin is a peptide hormone composed of 27 amino acids and is contained in cells found in the mucosa of the upper gastrointestinal tract, particularly in the gastric antrum, duodenum, and upper jejunum. Physiologic stimulation of secretin production occurs when gastric acid flows into the duodenum in response to food ingestion. The primary action of secretin is the promotion of water and bicarbonate secretion from the pancreas by affecting the activity of pancreatic acinar cells. In pancreatic juice cytology, a catheter is inserted into the main pancreatic duct to collect pancreatic juice, as well as cells detached from the ductal epithelium or tumor. Thereafter, a pathological diagnosis of malignant findings is made. In S-PJC, once pancreatic juice is aspirated from the main pancreatic duct, synthetic secretin is administered to promote the production of pancreatic juice, and the juice is collected again from the main duct. In other words, synthetic secretin allows the collection of pancreatic juice twice in a short period of time during ERCP. The increased pancreatic juice volume recovered from the catheter is expected to increase the number of cells to be collected. To stimulate a physiological process, such as food ingestion, during endoscopic procedures, secretin is loaded intravenously. Intravenous administration promotes synthetic secretin to promptly act on the pancreas; data from ERCP studies have shown an increase in pancreatic duct pressure within 1 min and almost complete relaxation within 5 min [8,9]. In our previous reports, pancreatic juice volume was also increased by loading synthetic secretin.

The aforementioned porcine secretin has not been marketed in Japan since 2004. A human synthetic secretin product manufactured using genetic modification technology approved by the U.S. Food and Drug Administration (FDA) has been manufactured and marketed for use in the United States since 2004. In the United States, synthetic secretin is licensed for use in (i) the pancreatic exocrine function test, (ii) the Zollinger-Ellison Syndrome test, and (iii) stimulation of pancreatic secretions to facilitate the identification of the ampulla of Vater and accessory papilla during endoscopic retrograde cholangiopancreatography (ERCP). On the other hand, in some countries, including Japan, secretin is not approved for clinical use. Furthermore, the unstable supply of secretin is also a problem. As its clinical usefulness becomes clearer, further mass production, distribution, and cost reductions are expected. According to the package insert for ChiRhoStim (ChiRhoClin Inc., Burtonsville, MD, USA), the adverse event information of synthetic secretin was 1.7% for nausea, 0.4% for flushing, and 0.5% for abdominal pain and vomiting [12]. Studies using synthetic secretin include secretin-enhanced magnetic resonance cholangiopancreatography (MRCP), which has been reported to increase visualization of the anatomy of the pancreatic duct and provide an adjunctive role in the diagnosis of conditions primarily of the pancreas [13]. There are still few publications on pancreatic juice cytology using synthetic secretin.

No studies have compared the techniques that may improve the diagnostic performance of PJC. Therefore, we retrospectively evaluated and compared the usefulness and safety of SPACE and S-PJC using a propensity score–matched analysis.

## 2. Materials and Methods

### 2.1. Study Design

This was a single-center retrospective observational study that was approved by the Institutional Review Board of the Tottori University Hospital in 2022 (registration number: 22A021). Biological samples were not collected for research purposes. The study subjects were informed of the outline of this study via the website or bulletin board and provided an opportunity to decline participation.

### 2.2. Eligibility Criteria

Patients who underwent S-PJC or SPACE for the pathological diagnosis of pancreatic masses between April 2010 and March 2012 at Tottori University Hospital were retrospectively enrolled. Patients who did not give consent and those with a surgically altered anatomy were excluded, except those who underwent a Billroth-I procedure.

### 2.3. Endoscopic Retrograde Pancreatography (ERP)

ERP was performed with a lateral viewing endoscope (JF260V, Olympus Optical Co., Ltd., Tokyo, Japan), cannula (M00535700, Boston Scientific Japan KK, Tokyo, Japan; or PR-110Q-1, Olympus Optical Co., Tokyo, Japan), and hydrophilic guidewire (M00556051 or M00556211; Boston Scientific Japan KK, Tokyo, Japan).

### 2.4. Synthetic Secretin and S-PJC

**Figure 1:**
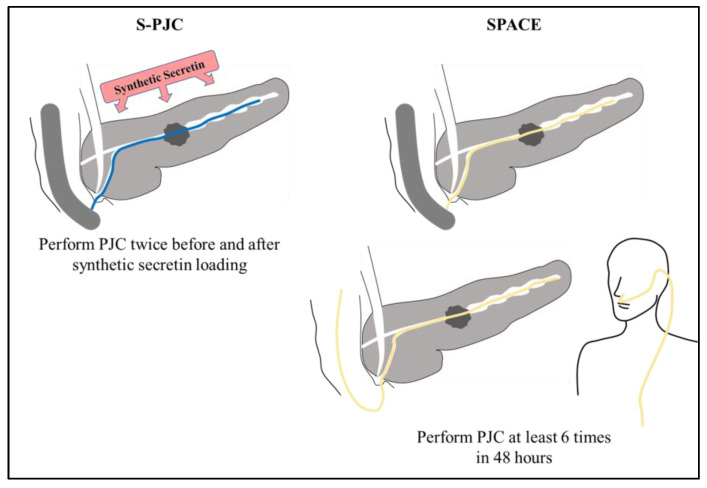
S-PJC and SPACE. Abbreviations: S-PJC, Secretin-loaded pancreatic juice cytology; SPACE, Serial pancreatic juice aspiration cytological examination; When performing S-PJC, PJC was performed twice during ERCP, before and after synthetic secretin loading. In SPACE, on the other hand, PJC s performed at least 6 times in 48 h.

ChiRhoStim (ChiRhoClin Inc., Burtonsville, MD, USA) was used as the synthetic secretin. First, pancreatic juice was aspirated for 5 min using a 10 mL syringe. Subsequently, 0.5 µg of synthetic secretin was intravenously loaded, and pancreatic juice was collected for another 5 min (Figure 1).

### 2.5. SPACE

A 5-Fr ENPD catheter (PD-PD5F (31SC) 260C12 [Gadelius Medical K.K., Tokyo, Japan]) was endoscopically placed into the main pancreatic duct. Up to 6 pancreatic fluid samples were collected over 1–2 days, and each sample was submitted for cytologic analysis. Thereafter, the ENPD catheter was removed at bedside (Figure 1).

### 2.6. Cytodiagnosis

PJC was performed by conventional Papanicolaou staining within two days of collecting pancreatic juice after ERCP, and class IIIb-V specimens were diagnosed as malignant. A professional pathologist (YH) diagnosed the aspirated specimens. The adequacy of the samples was evaluated based on the degree of denaturation and whether the volume of the samples was sufficient for diagnosis.

### 2.7. Follow-Up

The final diagnosis was confirmed by postoperative pathology or computed tomography imaging with at least one year of follow-up. Patients without malignant disease were followed up by imaging studies. All patients were carefully monitored for acute or delayed complications. Cotton’s diagnostic criteria were used to diagnose postendoscopic retrograde cholangiopancreatography pancreatitis (PEP) [14].

### 2.8. Examination Items

We evaluated the location, size and stenosis length of the lesion, specimen suitability, cytology results, final diagnosis with Union for International Cancer Control (UICC) stage classification (eighth edition), and procedure-related complications.

### 2.9. Propensity Score Matching

We performed propensity score matching to compensate for intergroup differences in the baseline characteristics of the study subjects that would affect the diagnostic accuracy of PJC for PDAC. For example, reports indicate that stenosis length [15] and location [16,17] affect the diagnostic performance of PJC for PDAC. Multivariate logistic regression models were used to calculate the propensity scores for performing S-PJC or SPACE. Moreover, we included the patients’ age, sex (male vs. female), lesion location (pancreatic head, body, or tail), and stenosis length in the model. Each patient in the S-PJC group was matched to a patient in the SPACE group using the nearest neighbor method, with a caliper range of 0.2 times the standard deviation of the pooled propensity score.

### 2.10. Statistical Analyses

We compared the diagnostic performances of the groups by *t*-test, Fisher’s direct test, and Cochran-Armitage test; a *p*-value of <0.05 was considered statistically significant. All statistical analyses were performed using EZR (Saitama Medical Center, Jichi Medical University, Saitama, Japan), a modified version of R commander (The R Foundation for Statistical Computing, Vienna, Austria, version 2.13.0) designed to add statistical functions frequently used in biostatistics [18].

## 3. Results

Table 1 lists all patients before and after propensity score matching; 75 patients were included in the analysis. Before propensity score matching, none of the characteristics differed between the SPACE and S-PJC groups. However, we found bias regarding stenosis length and disease. Following propensity score matching, we selected 26 patients who received SPACE and 26 patients who received S-PJC, and the characteristics were more balanced between the groups.

### 3.1. Diagnostic Capability

Table 2 summarizes the diagnostic performances of the PJC methods. In all cases, specimens were successfully collected for both methods. After propensity score matching, the sensitivity, specificity, and accuracy rates were 75.0%, 100%, and 92.3% for S-PJC, respectively, and 71.4%, 100%, and 92.3% for SPACE, respectively, meaning that there was no difference between the groups.

### 3.2. Complications

Four patients (15.4%) per group experienced complications. In the S-PJC group, three patients had PEP, and one had cholangitis. Two patients had PEP, and two had cholangitis in the SPACE group. The amylase levels one day after performing S-PJC or SPACE were 400.4 ± 370.9 U/L and 297.8 ± 386.1 U/L in the S-PJC and SPACE groups, respectively (*p* > 0.05) (Table 3).

## 4. Discussion

Chemotherapy for pancreatic ductal adenocarcinoma is associated with a variety of adverse events; thus, pretreatment diagnosis based on imaging findings alone is relatively risky. Therefore, the emphasis has traditionally been on pathological diagnosis before chemotherapy in patients with pancreatic ductal adenocarcinoma. On the other hand, in resectable cases, the need for preoperative pathology has been debated, as the resected specimen ensures a pathological diagnosis [19,20]. Some reports suggest that preoperative EUS-FNA does not affect the prognosis of patients with pancreatic cancer [19]. However, other studies suggest that needle tract seeding is possible during EUS-FNA for patients with cancer in the pancreatic body or tail, and physicians should consider this matter [20]. In cases in which pancreatoduodenectomy is planned for pancreatic head cancer, EUS-FNA is performed from the descending part of the duodenum, which is resected at the same time with the pancreatic head lesion during pancreaticoduodenectomy, meaning that residual recurrence is not feasible.

With this background, in recent years, the efficacy of preoperative chemotherapy for pancreatic cancer has been confirmed [21]. Preoperative chemotherapy requires the selection of an appropriate chemotherapy regimen based on the pathological diagnosis, even in patients scheduled for surgery where pathology specimens can be reliably obtained. Regardless of resectability, pretreatment pathology is now considered more important. Unless a diagnosis of the pretreatment pathology for PDAC can be obtained by EUS-FNA, alternative methods should complement the diagnostic performance. Given that the diagnostic performance of EUS-FNA is declining due to the procedure’s inability to avoid vessels and pancreatic ducts and to challenges associated with antithrombotic drug discontinuation, improvements in its accuracy may be difficult to achieve in the future. As an alternative method, PJC should continue to improve its accuracy. Reports also indicate that using EUS-FNA and PJC together improves diagnostic performance [22]. Thus, for difficult diagnoses, PJC should be considered in addition to repeated EUS-FNAs. However, the efficacy and safety of PJC still need to be fully elucidated before furthering the use of this technique in clinical practice.

Previous reports have evaluated the sensitivities of S-PJC and SPACE, reporting that PJC sensitivity was 38% for a single collection, increasing to 75% with SPACE [23]. S-PJC sensitivity has also improved from 50.9% to 74.0% with synthetic secretin. Furthermore, 13 cases of PDAC undiagnosable by EUS-FNA were pathologically diagnosed [24]. Previous reports have shown that positive cytological results were more frequent in patients with PDAC of the pancreas head than in those of the pancreas body or tail [15,25]. In this study, after propensity score matching, the association between the location of the lesion and the diagnostic accuracy was examined, but no consistent trend was observed. Further studies with a larger number of patients are needed to clarify how lesion location affects both detection methods. Factors that influenced the positive diagnosis were examined, but no significant differences in gender, age, location, or tumor diameter were observed. Mie et al. aimed to improve diagnostic performance by combining SPACE with brush cytology, but its effectiveness was limited [26]. Although SPACE and S-PJC have improved diagnostic performance compared to single PJC, no studies have compared the diagnostic performance between the two techniques. In this study, we found that the diagnostic performances of S-PJC and SPACE were comparable.

Both patients who developed PEP in the SPACE group had an IPMN. In the S-PJC group, one patient had IPMN, one patient had PDAC, and one patient had a pancreatic neuroendocrine neoplasm. Previous studies reported SPACE-related PEP complications in 4.1–12.4% of all patients [21,24]. Mouri et al. reported that SPACE was 4.1 times more likely to induce PEP in patients with IPMN than in patients without IPMN [27]. Additionally, Ito et al. found that pancreatic duct stenting in patients with IPMN was a risk factor for PEP (odds ratio, 2.9; 95% confidence interval, 1.2, 7.1) [28]. These results suggest that SPACE may be too aggressive for PDAC patients with IPMN. On the other hand, S-PJC has been reported to be associated with PEP in 8.7% of patients [24]. There are no reports examining whether the presence or absence of IPMN makes a difference in the complication rate of PEP after S-PJC. However, the short procedure time may have an advantageous effect. Most reports of PEP after PJC are mild and improve with conservative treatment, but severe PEP does occur, albeit rarely. Therefore, efforts to reduce the risk of PEP should continue.

Efficacy and safety evaluations alone provide an incomplete comparison of the two techniques. The third factor is the burden on the patient and the medical staff. For SPACE to be performed, an ENPD catheter is placed during ERCP, and pancreatic fluid is collected an average of six times over a period of 1–2 days. The ENPD catheter size of 4Fr has also been used since 2016. Mouri et al. reported that SPACE performed with a 4Fr catheter had fewer complications than with a 5Fr catheter [27]. Both sizes of ENPD catheters (Gadelius Medical K.K., Tokyo, Japan) used in that study were made of polyurethane and curved from the papilla to the duodenal bulb. The catheter section inserted into the pancreas was 10 cm long and had a slight S shape. During the placement period, patients will feel the foreign body of a 4-Fr or 5-Fr catheter from the external nasal cavity to the pharynx, larynx, and esophagus. Medical staff also need to go to the bedside of admitted patients for multiple pancreatic fluid collections, collect the fluid using a syringe, and submit it to the pathology department each time. This is because prompt sample processing is important in PJC, which is diagnosed using denatured cells. The morphologies of cells in pancreatic juice are often difficult to determine because they have been exfoliated for a long time and are strongly denatured by digestive enzymes. On the other hand, when S-PJC is performed, the ENPD catheter is not placed, and pancreatic fluid collection is completed during ERCP. A previous report indicated that the administration of secretin does not cause adverse events [24,29]. The underlying problem is that obtaining a stable supply of synthetic secretin for S-PJC is difficult, as secretin administration for pancreatic fluid collection is not approved in most countries.

As for long-term progress after the PJC, early-stage PDAC may develop iatrogenic recurrence postoperatively [30], requiring the most attention during follow-up. Moreover, PDAC cells have been reported to implant into the pancreatic ductal epithelium [31]. The duration from PDAC cells to mass is 11.8 years for intraepithelial carcinoma and 6.8 years for intraepithelial to invasive carcinoma [32]. These reports make it abundantly clear that iatrogenic recurrence can occur after early-stage PDAC resection. During follow-up after PDAC resection, if the diameter of the main pancreatic duct with jejunal anastomosis is dilated and ectopic recurrence is suspected, PJC should be performed again. However, the stable placement of an ENPD in the reconstructed intestinal canal postoperatively can be difficult, and S-PJC may be preferred.

The study has some limitations. This was a single-center, retrospective study that included a very small number of cases; thus, this study has some selection bias. Furthermore, the number of endoscopists was limited, and only one pathologist (YH) performed diagnosis. All SPACEs were performed using 5Fr ENPD catheters, not 4Fr ENPD catheters. Nonsteroidal anti-inflammatory drugs, intravenous fluids, pancreatic stents, epinephrine spraying, or combinations of these have been reported to be useful in preventing PEP [33,34]. A recent meta-analysis found that 100 mg of diclofenac was effective in preventing PEP [35]. However, none of these procedures were used in the present study because there was no evidence of their efficacy during the study period. Moreover, notably, synthetic secretin has not been approved by the Pharmaceutical Affairs Law in Japan, and it was imported after obtaining approval from our hospital’s Ethics Review Committee. Therefore, availability may be a key challenge for other researchers wishing to conduct similar studies in Japan.

## 5. Conclusions

Patients with PDAC have a poor prognosis, and a qualified diagnosis by pathological findings is important to rapidly decide the appropriate treatment strategy. Since some PDACs are difficult to be diagnosed by EUS-FNA, PJC will continue to be in demand as a pathological diagnostic method. SPACE is a very useful diagnostic method that is widely performed, especially in Japan, and is expected to continue to develop in the future. S-PJC is performed in a limited number of centers due to problems such as an unstable supply of synthetic secretin. Nevertheless, it is highly useful, at least in cases of IPMN and postoperative bowel reconstruction. Furthermore, S-PJC is less burdensome than SPACE for patients, and we did not find any difference in diagnostic performance or procedural safety between S-PJC and SPACE in this study. 

## Figures and Tables

**Table 1:** Patient characteristics before and after propensity score matching.

		All Patients			Propensity Score-Matched Patients		
		S-PJC	SPACE	*p* Value	S-PJC	SPACE	*p* Value
n		49	26		26	26	
**Age**	(S.D.)	69.18 (±8.26)	70.08 (±13.19)	0.72	70.12 (±8.48)	70.08 (±13.19)	0.99
**Sex (%)**	Male	31 (63.3)	19 (73.1)	0.449	18 (69.2)	19 (73.1)	1
	Female	18 (36.7)	7 (26.9)		8 (30.8)	7 (26.9)	
**Final** **diagnosis (%)**	PDAC	22 (44.9)	7 (26.9)	0.166	8 (30.8)	7 (26.9)	0.557
	CP	7 (14.3)	2 (7.7)		4 (15.4)	2 (7.7)	
	IPMN/MCN	14 (28.6)	15 (57.7)		11 (42.3)	15 (57.7)	
	AIP	4 (8.2)	2 (7.7)		1 (3.8)	2 (7.7)	
	PNEN	2 (4.1)	0 (0.0)		2 (7.7)	0 (0.0)	
**Location (%)**	Ph	23 (46.9)	9 (34.6)	0.713	10 (38.5)	9 (34.6)	1
	Pb	14 (28.6)	10 (38.5)		9 (34.6)	10 (38.5)	
	Pt	9 (18.4)	6 (23.1)		6 (23.1)	6 (23.1)	
	Diffuse	3 (6.1)	1 (3.8)		1 (3.8)	1 (3.8)	
**TNM classification**							
**PDAC Stage**	0/IA/IB/IIA/IIB/III/IV	0/1/3/1/2/5/10	1/0/1/0/3/0/2		0/1/1/0/0/3/3	1/0/1/0/3/0/2	
**Stenosis length**	(mm)	16.19 (±16.36)	8.97 (±12.67)	0.054	7.63 (±11.78)	8.97 (±12.67)	0.696

Abbreviations: S-PJC, Secretin-loaded pancreatic juice cytology; SPACE, Serial pancreatic juice aspiration cytological examination; PDAC, pancreatic ductal adenocarcinoma; CP, Chronic pancreatitis; IPMN, Intraductal papillary mucinous neoplasm; MCN, Mucinous cystic neoplasm; AIP, Autoimmune pancreatitis; and PNEN, Pancreatic neuroendocrine neoplasm. The bias regarding stenosis length and final diagnostic disease are statistically matched after propensity-score matching. Twenty-six patients were selected for each of the S-PJC and SPACE arms.

**Table 2:** Diagnostic performances of the PJC methods before and after propensity score matching.

Data before Propensity Score Matching												
	Adequate Specimen		Sensitivity		Specificity		PPV		NPV		Accuracy	
	(%)		(%)		(%)		(%)		(%)		(%)	
**S-PJC**	100	(49/49)	59.1	(13/22)	100	(27/27)	100	(13/13)	75	(27/36)	81.6 ^†^	(40/49)
**SPACE**	100	(26/26)	71.4 *	(5/7)	100	(19/19)	100	(5/5)	90.5	(19/21)	92.3 ^†^	(24/26)
*^, †^: Fisher’s test; *p* value = 0.947, 0.164												
**After Propensity Score Matching**												
	**Adequate** **Specimen**		**Sensitivity**		**Specificity**		**PPV**		**NPV**		**Accuracy**	
	(%)		(%)		(%)		(%)		(%)		(%)	
**S-PJC**	100	(26/26)	75 *	(6/8)	100	(18/18)	100	(6/6)	90	(18/20)	92.3 ^†^	(24/26)
**SPACE**	100	(26/26)	71.4 *	(5/7)	100	(19/19)	100	(5/5)	90.5	(19/21)	92.3 ^†^	(24/26)
*^, †^: Fisher’s test; *p* value = 0.662, 1												

Abbreviations: S-PJC, Secretin-loaded pancreatic juice cytology; SPACE, Serial pancreatic juice aspiration cytological examination; PPV, Positive predictive value; and NPV, Negative predictive value. After propensity score matching, the sensitivity, specificity, and accuracy rates were 75.0%, 100%, and 92.3% for S-PJC, respectively, and 71.4%, 100%, and 92.3% for SPACE, respectively.

**Table 3:** Safety of PJC methods before and after propensity score matching.

	All Patients			Propensity Score-Matched Patients		
	S-PJC	SPACE	*p* Value	S-PJC	SPACE	*p* Value
n	49	26		26	26	
**AMY value on the next morning (U/L)**	332.4 (±320.7)	297.8 (±386.1)	NS *	400.4 (±370.9)	297.8 (±386.1)	0.333 ^†^
**Adverse Events**	6 (12.2%)	3 (11.5%)	0.776	4 (15.4%)	3 (11.5%)	1
**PEP**	5 (10.2%)	2 (7.7%)	0.951	3 (11.5%)	2 (7.7%)	1
**Cholangitis**	1 (2.0%)	2 (7.7%)	0.771	1 (3.8%)	2 (7.7%)	1

*, Cochran-cox; ^†^, *t*-test; Abbreviations: S-PJC, Secretin-loaded pancreatic juice cytology; SPACE, Serial pancreatic juice aspiration cytological examination; AMY, Serum amylase value; and PEP, postendoscopic retrograde cholangiopancreatography pancreatitis. After propensity score matching, there was no difference in the frequency of complications.

## Data Availability

The datasets generated during and/or analyzed during the current study are available from the corresponding author upon reasonable request.
